# Biochemical and Structural Characterization of Mycobacterial Aspartyl-tRNA Synthetase AspS, a Promising TB Drug Target

**DOI:** 10.1371/journal.pone.0113568

**Published:** 2014-11-19

**Authors:** Sudagar S. Gurcha, Veeraraghavan Usha, Jonathan A. G. Cox, Klaus Fütterer, Katherine A. Abrahams, Apoorva Bhatt, Luke J. Alderwick, Robert C. Reynolds, Nicholas J. Loman, VijayaShankar Nataraj, Carlos Alemparte, David Barros, Adrian J. Lloyd, Lluis Ballell, Judith V. Hobrath, Gurdyal S. Besra

**Affiliations:** 1 School of Biosciences, University of Birmingham, Edgbaston, Birmingham, B15 2TT, United Kingdom; 2 Department of Chemistry, University of Alabama at Birmingham, College of Arts and Sciences, 1530 3rd Avenue South, Birmingham, Alabama, 35294-1240, United States of America; 3 Diseases of the Developing World, GlaxoSmithKline, Severo Ochoa 2, 28760, Tres Cantos, Madrid, Spain; 4 Department of Life Sciences, University of Warwick, Coventry, CV4 7AL, United Kingdom; 5 Organic Chemistry Department, Southern Research Institute, Birmingham, Alabama, 35205, United States of America; University of Delhi, India

## Abstract

The human pathogen *Mycobacterium tuberculosis* is the causative agent of pulmonary tuberculosis (TB), a disease with high worldwide mortality rates. Current treatment programs are under significant threat from multi-drug and extensively-drug resistant strains of *M. tuberculosis*, and it is essential to identify new inhibitors and their targets. We generated spontaneous resistant mutants in *Mycobacterium bovis* BCG in the presence of 10× the minimum inhibitory concentration (MIC) of compound **1**, a previously identified potent inhibitor of mycobacterial growth in culture. Whole genome sequencing of two resistant mutants revealed in one case a single nucleotide polymorphism in the gene *aspS* at ^535^GAC>^535^AAC (D179N), while in the second mutant a single nucleotide polymorphism was identified upstream of the *aspS* promoter region. We probed whole cell target engagement by overexpressing either *M. bovis* BCG *aspS* or *Mycobacterium smegmatis aspS,* which resulted in a ten-fold and greater than ten-fold increase, respectively, of the MIC against compound **1**. To analyse the impact of inhibitor 1 on *M. tuberculosis* AspS (Mt-AspS) activity we over-expressed, purified and characterised the kinetics of this enzyme using a robust tRNA-independent assay adapted to a high-throughput screening format. Finally, to aid hit-to-lead optimization, the crystal structure of *apo M. smegmatis* AspS was determined to a resolution of 2.4 Å.

## Introduction

The causative agent of tuberculosis (TB), *Mycobacterium tuberculosis*, accounts for nearly 1.4 million fatalities worldwide, with an incidence rate of 8.8 million cases *per* annum [1]–[3]. This situation is compounded due to co-infection with the Human Immunodeficiency Virus (HIV), and the rise in infections with multi-drug resistant TB (MDR-TB) [4], extensively-drug resistant TB (XDR-TB) and totally-drug resistant (TDR-TB) strains [5]. High Throughput Screening (HTS) of extensive compound libraries now enables researchers to focus on whole cell phenotypic profiling of hits with bactericidal or bacteriostatic activity rather than limiting efforts to single-enzyme methodologies [6]–[11]. In this regard, the discovery of the inhibitor diarylquinoline TMC207 (Bedaquiline) was a notable success where HTS was coupled with the use of whole genome sequencing (WGS) of spontaneous resistant mutants to identify the cellular target, *M. tuberculosis* ATP synthase [12]–[15]. In addition, mode of action studies of other anti-TB compounds, such as SQ109, BM212, adamantyl ureas, benzimidazole, BTZ, TCA, and imidazo[1,2-*a*]pyridine related derivatives [16]–[24] have established their cellular targets by WGS of spontaneous resistant mutants generated against these compounds [25].

**Figure 1:**
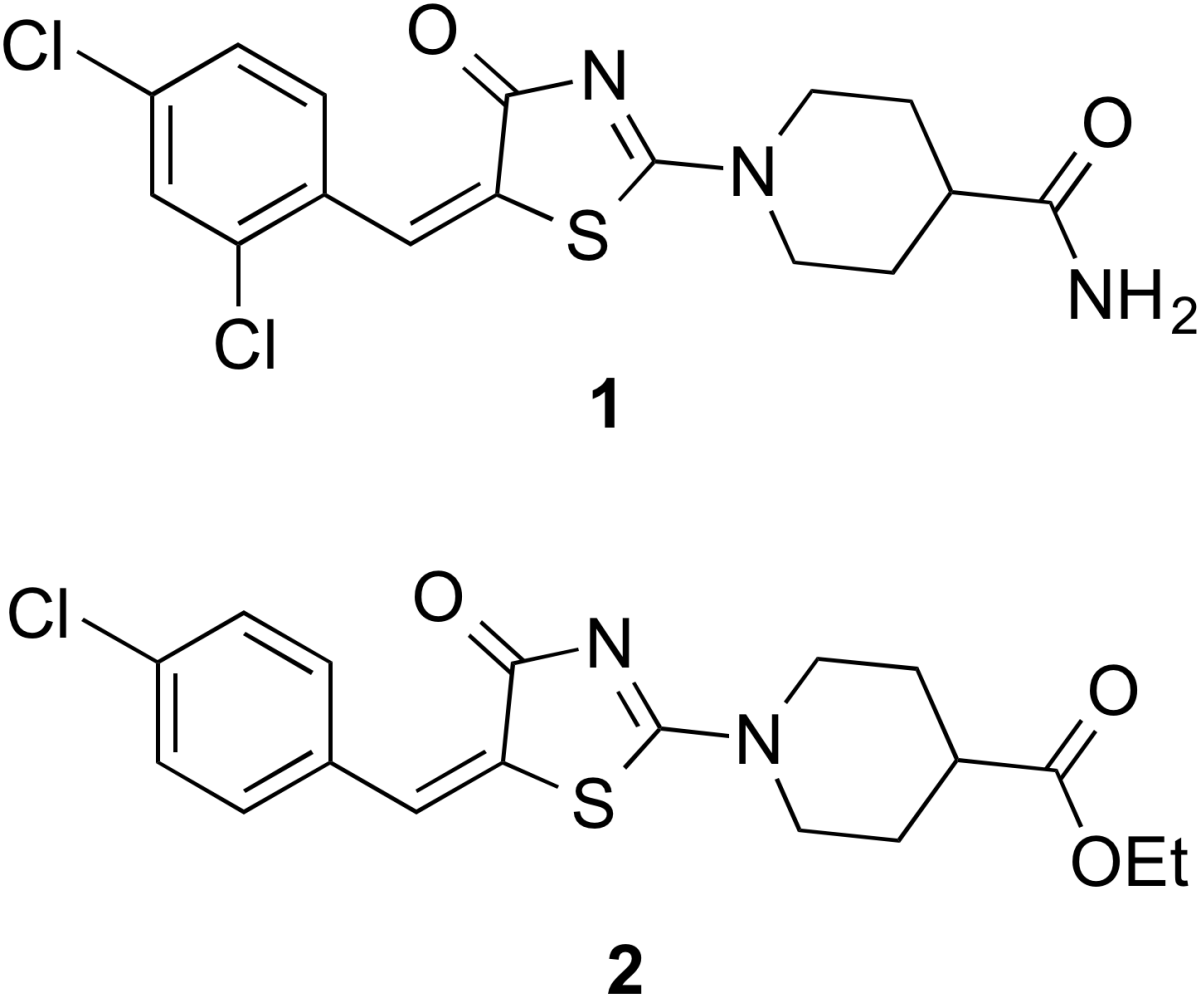
Structures of compounds 1 and 2 used in this study.

Using this established strategy of target deconvolution, *M. tuberculosis* aspartyl tRNA synthetase *aspS* has been recently identified as the cellular target of compound **1** (**Figure 1**) [26]. The aspartyl tRNA synthetase (*aspS*) belongs to a group of twenty aminoacyl tRNA synthetases (aaRSs) which are divided into two classes, class I and class II, which are differentiated by their aminoacylation domains [27]. Aminoacylation by aaRSs occurs in a two-step process: (i) activation of the amino acid with ATP resulting in the formation of an aminoacyl-adenylate, and (ii) aminoacylation of the tRNA by the aminoacyl adenylate [28]. Several prokaryotes including mycobacteria do not contain the open reading frame encoding the asparagine tRNA synthetase and the glutamine tRNA synthetase [29], [30]. AspS and Glutamyl tRNA synthetase (GluS) play the role of a non-discriminating amino acyl tRNA synthetase and could also acylate tRNA^Asn^ and tRNA^Gln^ with Aspartate and Glutamate respectively [29]. The eighteen aaRSs of mycobacteria are essential for protein synthesis and represent viable targets for the development of new therapeutics [29], [31]. Indeed, several naturally occurring antimicrobials have been shown to be specific aaRSs inhibitors [32]. Among them are Microcin C (McC) produced by Enterobacteriaceae, which inhibits translation by preventing aminoacylation of tRNA^Asp^ by AspS and Tobramycin, an aminoglycoside competitive inhibitor with respect to tRNA^Asp^ [33]–[35]. McC functions through a trojan horse mechanism because it enters the bacterial membrane as a prodrug by facilitated transport, undergoes processing by cellular aminopeptidases and releases a toxic moiety called aspartyl adenylate which inhibits AspS [33], [36]. Herein, we report the detailed biochemical characterization of the *M. tuberculosis* aspartyl-tRNA synthetase AspS and we report the 2.4 Å-resolution crystal structure of the *M. smegmatis* homologue.

## Materials and Methods

### Ethics Statement

All experiments were approved by the University of Birmingham and Diseases of the Developing World (DDW-GSK) ethical committee where required and there are no ethical issues to report.

### Chemicals & Materials

Phusion DNA polymerase, DNA restriction enzymes and T4DNA ligase, One Taq hot start 2X master mix RT-PCR kit, and RNase free DNase set were procured from New England Biolabs (NEB). RNeasy mini kit was obtained from Qiagen. Trizol reagent and Turbo DNA-free kit were obtained from Life Technologies. β, γ adenylyl imidodiphosphate lithium salt (ADPNP), Adenylylmethylenediphosphonate disodium salt (ADPCP), ATP, NADP^+^, tetrasodium pyrophosphate, and CHAPS were purchased from Sigma Aldrich (St. Louis, MO, USA). Compounds **1** (F1673-0402) and **2** (6632725) (**Figure 1**) were purchased from Lifechemicals.com and Chembridge, respectively and were used as such. A mixture of yeast hexokinase and *Leuconostoc mesenteroides* glucose-6-phosphate dehydrogenase was obtained from Roche Applied Science, Mannheim, Germany. Oligonucleotide primers were synthesised by MWG Eurofins, Germany.

### Generation and WGS of spontaneous resistant mutants, cloning of *M. bovis* BCG and *M. smegmatis aspS* in pMV261 and MIC determination of compound 1 and 2

Spontaneous resistant mutants of *M. bovis* BCG were generated against compound **1** at 10× MIC (MIC = 2.6 µM) by plating 10^8^ cells on solid media as described previously [23], [26]. Purified genomic DNA from each mutant (two) and the parental strain were prepared for sequencing using the Illumina Nextera DNA Sample Preparation Kit (Illumina, Great Chesterford, UK). Genomic DNA (25 ng) was simultaneously fragmented and tagged with adapters using the Nextera transposome. The tagged fragments were amplified by limited-cycle PCR, incorporating Illumina sequencing primer sequences and indices required for cluster generation and sequencing. The DNA libraries were purified using Agencourt AMPure XP beads (Beckman Coulter Genomics, High Wycombe, UK) and quantified with Quant-iT PicoGreen dsDNA kit (Life Technologies). The median fragment size (420–575 bp) of the final libraries was determined on an Agilent Technologies 2100 Bioanalyzer using a High Sensitivity DNA chip. The libraries were diluted to 2 nM, combined and denatured according to the MiSeq preparation guide. The libraries were sequenced on a MiSeq Benchtop Sequencer using the MiSeq Reagent Kit v2, 300 cycles. Reads were aligned to the *M. bovis* BCG Pasteur 1173P2 reference genome sequence (accession: NC_008769.1) using the Burrows-Wheeler Aligner (BWA) version 0.6.1 in bwasw mode and default settings [37]. Putative variants were then detected using samtools mpileup pipeline using default settings. The output was filtered with VarScan v2.3.5 [38], [39] to detect variants with an allele frequency of greater or equal to 90% with more than five covering reads and with a p-value of less than 0.005. The putative effect of SNPs on coding sequences was determined using snpEff 2.0.5d [40].

*M. bovis* BCG *aspS* and *M. smegmatis aspS* were amplified by PCR using the oligonucleotides shown in **Table 1**. PCR was carried out using Phusion DNA polymerase and as *per* the conditions recommended by the manufacturer. The PCR product was digested with BamHI and HindIII and ligated into plasmid pMV261 which was similarly cut and transformed into *E. coli* Top 10 cells. The sequences of the cloned constructs were confirmed by sequencing. The plasmids were transformed by electroporation into electrocompetent *M. bovis* BCG cells for MIC determination against compounds **1** (F1673-0402, Lifechemicals.com) and **2** (6632725, Chembridge) (**Figure 1**), in comparison to an empty pMV261 control, as reported previously [23], [26].

**Table 1:** Primers used in this study.

Primer	Sequence (5′ to 3′)
BCG *aspS*-pMV261 forward	GATCGATCGGATCCAGTGTTTGTGCTGCGCAGCCACGCC
BCG *aspS*-pMV261 reverse	GATCGATCAAGCTTCTATTTATTCACACTCGGGTGCGAC
*M. smegmatis aspS*-pMV261 forward	GATCGATCGGATCCAGTGCTGCGCACTCATGCCGCCGG
*M. smegmatis aspS*-pMV261 reverse	GATCGATCAAGCTTTCACGCCTTTGACTTGGCGTCTTC
*M. tuberculosis aspS*-pET28b forward	GATCGATCCATATGTTTGTGCTGCGCAGCCAC
*M. tuberculosis aspS*-pET28b reverse	GATCGATCAAGCTTTGCCTGCTGGACCCGCTTG
*M. smegmatis aspS*-pET28b forward	GATCGATCCATATGCTGCGCACTCATGCCGCC
*M. smegmatis aspS*-pET28b reverse	GATCGATCAAGCTTCGCCTTTGACTTGGCGTC

### Isolation and purification of total RNA, cDNA synthesis and RT-PCR

The wild type *M. bovis* BCG, promoter up mutant and the AspS mutant cultures (50 ml each) were grown until mid logarithmic phase (A_600_ of 0.8) and the total RNA was extracted from the pellet for cDNA synthesis using the RNA-Trizol protocol as described [41], [42]. The isolated RNA was purified using RNeasy spin columns (Qiagen), digested with RNase free DNase (NEB) and the RNA cleaned up using the RNeasy spin column to remove DNase. The residual amount of genomic DNA contamination was removed by treating the RNA again with Turbo DNA free kit DNase (Life Technologies) and reagents removed using the same kit to remove DNase and divalent cations from the RNA. The purified RNA samples (600 ng each) were used as templates for cDNA synthesis using the NEB kit as per manufacturers instructions. Control reactions were set up without addition of reverse transcriptase. *M. bovis* BCG *aspS* gene specific primers were designed to amplify by RT-PCR the 200 bp intergenic region. The oligos designed for RT-PCR are *M. bovis* BCG *aspS* Left (5′-3′)CGATCTCCGAGGAAGTTCTG and *M. bovis* BCG *aspS* Right (5′-3′) ACATACGGTGCCTGGAAGAC, respectively. PCR was carried out as per the manufacturers instructions using the master mix provided in the One Taq hot start 2X master mix kit (NEB), *M. bovis* BCG *aspS* specific primers and the synthesised cDNA as template. The RT-PCR cycling conditions were programmed for 25, 30, 40 and 50 cycles, respectively, to get sufficient yields of the 200 bp amplified product. The transcripts (20 µl each) were analysed on a 2% agarose gel.

### Cloning of *M. tuberculosis* and *M. smegmatis aspS* in pET28b

The *M. tuberculosis* and *M. smegmatis aspS* were amplified by PCR from *M. tuberculosis* H37Rv and *M. smegmatis* genomic DNA, respectively, and cloned into pET28b to generate C-terminally-tagged Histidine (His_6_) fusion proteins. The oligonucleotide primers used for pET28b cloning are shown in **Table 1**. The forward primer contains the NdeI restriction site and the reverse primer contains the HindIII restriction site. The PCR product was purified, digested with NdeI and HindIII and ligated into the expression vector pET28b using NdeI and HindIII restriction sites. Both the C-terminal His_6_ tagged constructs were verified by sequencing.

### Expression and purification of the C-terminally His_6_-tagged Mt-AspS for activity assays

The pET28b-Mt-*aspS* construct with the C-terminal His_6_-tag was transformed into *E. coli* C41 (DE3) cells for protein expression. An overnight starter culture was prepared by inoculating a single colony from a freshly transformed plate into LB broth containing kanamycin (25 µg/ml). A 1% inoculum of the starter culture was used to inoculate 1 L of LB broth containing kanamycin (25 µg/ml), grown at 37°C until the OD_600_ of 0.6 was attained, induced with 1 mM isopropyl-β-D-thiogalactopyranoside (IPTG), and cultured at 16°C for 18 hours. The harvested cells were resuspended in lysis buffer containing 20 mM Tris-HCl pH 8.0, 500 mM NaCl, 10% glycerol, 40 mM imidazole, 1 mg/ml lysozyme and protease inhibitor tablet and disrupted by sonication with eight cycles of 30 s pulses at 30 s intervals. The crude lysate was clarified by centrifugation at 15,000 rpm for 45 minutes. The supernatant was loaded onto a pre-packed Ni^2+^ Sepharose HisTrap high performance column (GE Healthcare), which was equilibrated with buffer A (lysis buffer without lysozyme and protease inhibitor). The column was washed with buffer A and eluted with a stepwise gradient of imidazole (50, 75, 100, 250 and 500 mM) in Buffer A. The purified fractions were analysed by SDS-PAGE and the relevant fractions containing Mt-AspS were pooled and dialysed against Buffer B (20 mM Tris-HCl pH 8.0, 50 mM NaCl, 10% glycerol, 1 mM DTT and 100 µM EDTA) and concentrated with an Amicon ultrafiltration unit containing a 10 kDa cut off membrane. Protein concentration was measured with the BCA protein reagent kit using BSA as a standard.

### Mt-AspS *in vitro* activity assay

The tRNA-independent pyrophosphate exchange assay, as described previously [43], [44] was adapted to a 96-well microtitre plate format, for monitoring the reactions. ADPNP + **L**-Asp → AMP-Asp + PNP and AMP-Asp + *PP*_i_ → ATP + **L**-Asp. The AspS activity assays were performed in triplicate at 37°C for 30 minutes. The assay was modified from that reported previously [44] as follows: the reaction mixture for the Mt-AspS assay was in a final assay volume of 200 µl containing 20 mM HEPES pH 7.6, 4 mM MgCl_2_, 50 mM KCl, 1 mM DTT, 2 mM ADPCP or 3 mM ADPNP, 10 mM **D**-glucose, 0.5 mM NADP^+^, 10 mM **L**-Asp, 3 µg of yeast hexokinase and *L. mesenteroides* glucose-6-phophate dehydrogenase mixture, 6 µg of Mt-AspS and 250 µM of pyrophosphate. Using 96-well Costar 3603 black plates with a clear bottom, the assay was initiated by the addition of pyrophosphate using the injector in the plate reader. The plates were shaken for a few seconds and absorbance was measured at 340 nm with a Pherastar FS microtitre plate reader (BMG Labtech). Initial reaction rates for each substrate (i.e. ADPNP or ADPCP or **L**-Asp or *PP_i_*) were measured at 10 to 12 different substrate concentrations, while keeping the other two substrates at a fixed saturating concentration. K*_M_* values were determined by non-linear least square fitting of Michaelis-Menten curves and expressed as mean +/− standard error of mean. Assays were repeated in 96-well Greiner flat bottom black plates, measuring fluorescence intensity with excitation and emission wavelength of 350 and 450 nm, respectively. The Mt-AspS kinetic data was analysed using Mars software and exported to Excel with graphs generated using graphPad prism version 5.

### Hexokinase/Glucose-6-phosphate dehydrogenase (HK/G6P-DH) coupled assay

Hexokinase phosphorylates glucose to glucose-6-phosphate, which in turn is coupled to glucose-6-phosphate dehydrogenase, which oxidises glucose-6-phosphate to gluconate-6-phosphate in the presence of the cofactor NADP^+^. The reaction mixture consisted of 20 mM Hepes pH 7.6, 4 mM MgCl_2_, 1 mM DTT, 0.1 mM ATP, 50 mM KCl, 0.5 mM NADP^+^, 10 mM glucose and 120 ng of yeast hexokinase and *L. mesenteroides* glucose-6-phophate dehydrogenase mixture in a total volume of 200 µl. The ATP concentration was varied from 20 to 450 µM to determine the K*_M_* for ATP. The reaction was initiated with **D**-glucose and the rate of reduction of NADP^+^ to NADPH was measured in the absorbance mode at 340 nm and assays were performed in triplicate.

### IC_50_ determination of compound 1 and 2

The IC_50_ dose response assays for **1** and **2** were done at the apparent K*_M_* of ADPCP and **L**-Asp, 24 µg of Mt-AspS and 250 µM PP*_i_*. All compounds were dissolved in DMSO. In the maximum (100% activity) and minimum (0% activity) controls similar volumes of DMSO were added instead of compounds **1** and **2**. DMSO was used previously at a concentration of 10% to screen a library of compounds in a tRNA-independent assay of aminoacyl tRNA synthetases, and CHAPS had been used at 1.5% in the MurM enzymatic assay [45]. The highest concentration of DMSO and CHAPS used in the Mt-AspS assay were 10% (v/v) and 1.5% (w/v), respectively. Different concentrations of **1** and **2** were aliquoted initially into the wells of a microtitre plate followed by the reaction mixture and the reaction initiated with PP*_i_* and read in the fluorescence mode using Pherastar. The controls were run with Mt-AspS (Maximum) and without Mt-AspS (Minimum) for each compound. Percentage activity was calculated using the formula: [Activity of enzyme with test compound – Minimum/Maximum-Minimum] × 100, where Maximum is the activity in the presence of enzyme, substrates and cofactors, numerator *Minimum* is the activity in the presence of substrates, cofactors and test compound but without enzyme and denominator *Minimum* is the background activity in the presence of substrates and cofactors but without enzyme. IC_50_ was defined as the concentration resulting in 50% inhibition of enzymatic activity. The dose response curves of the inhibitors were plotted as the percentage activity *versus* the log concentration of the inhibitor and the IC_50_ values were determined using non-linear regression function in GraphPad Prism.

### X-ray crystallographic structure determination of *M. smegmatis* AspS (Ms-AspS)

Prior to setting up crystallisation experiments, protein was dialysed into 20 mM Tris-HCl pH 8.0, 50 mM NaCl, 10% glycerol, 100 µM EDTA and 1 mM DTT and concentrated by ultrafiltration. Crystals of Ms-AspS were obtained by vapour diffusion in 96-well sitting drop plates (SwissSci), using a Mosquito liquid handler (TTP Labtech) to pipette droplets of 100 nl Ms-AspS (25 mg/ml) +100 nl reservoir solution. Initial crystals were obtained from commercial sparse matrix screens (Molecular Dimensions) grown over a reservoir of 30% (v/v) pentaerythritol ethoxylate, 0.1 M magnesium formate, 0.1 M Tris-HCl, pH 8.5. Crystals were briefly immersed in reservoir solution complemented with 15% glycerol for cryoprotection, mounted in nylon loops and flash frozen in liquid nitrogen. X-ray diffraction data was recorded on a MicroMax 007HF X-ray generator equipped with a Saturn 944 CCD detector (Rigaku), integrated and scaled using XDS, XSCALE [46]. Initial phases were obtained by molecular replacement (PHASER, search model pdb entry 1C0A) [47]. The model was rebuilt and refined [48], [49]. Coordinates and structure factors for Ms-AspS are deposited in the Protein Data Bank (www.rcsb.org).

### *In silico* methods

Prior to docking, the *M. smegmatis* AspS crystal structure was refined applying Prime preparation and refinement tools of the Protein preparation wizard implemented in the Schrödinger software package. After the addition of hydrogens and detection of disulfide bonds the structure was optimized by applying default parameters of the Impref utility using the OPLS2001 force field. The maximum allowed root-mean-square deviation between the refined structure and the input crystal structure was 0.3. The same refinement protocol was applied to the D174N and T565I point mutant AspS structures following mutation of the respective residue. The ligand structure was prepared using the LigPrep utility at pH7.4. For docking runs the Induced Fit method [50] implemented in the Schrödinger software was applied, combining the Glide docking method combined with Prime structural refinement tools to account for the flexibility of protein side chains within 5 Å of the ligand during docking. Induced Fit settings were at default values except the size of the box that encloses the targeted site was set to 22 Å and extra-precision mode was selected. In the Induced Fit workflow the initial Glide docking run used softened potentials (Van der Waals scaling of 0.50), followed by refinement of the structures applying Prime side chain optimization of residues within 5 Å from docked ligand poses. The derived ‘induced-fit’ receptor structures were then utilized for the final step of Glide re-docking with default parameters, applied to structures within 30 kcal/mol of the lowest energy structure.

## Results and Discussion

### Target identification and whole cell target engagement for compound 1 and 2 against *M. bovis* BCG

**Figure 2:**
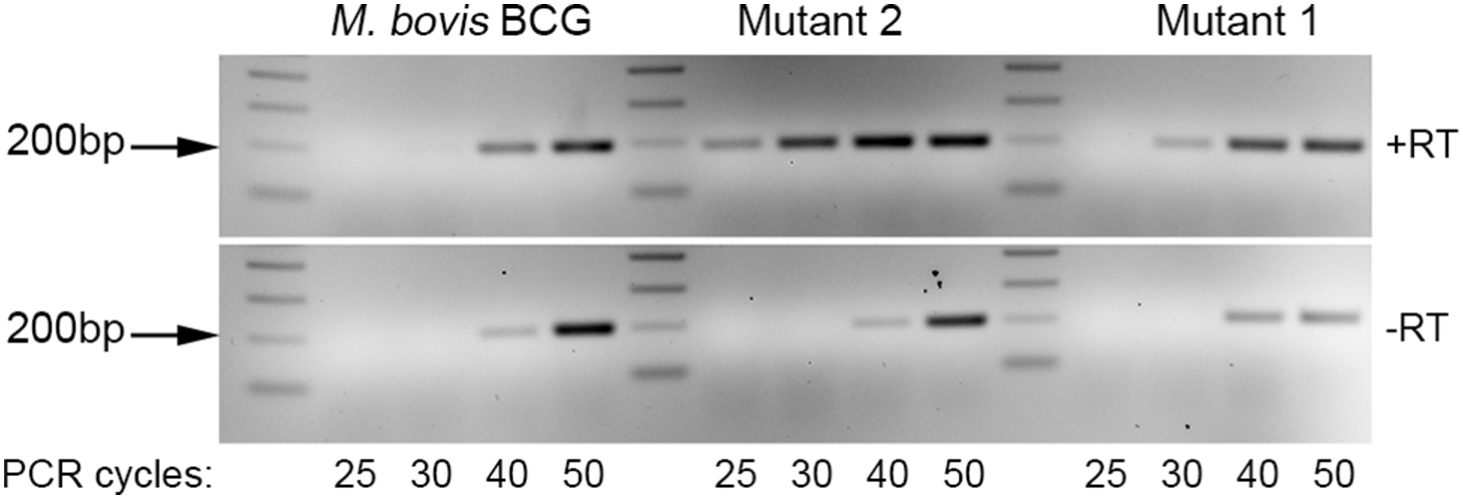
Comparison of the expression levels of the transcript produced by wild type *M. bovis* BCG, *aspS* mutant 1 and promoter up mutant 2 by RT-PCR. RT-PCR was performed with RT (upper panel) and without RT (lower panel). The RT-PCR cycling conditions were programmed for 25, 30, 40 and 50 cycles, respectively, to get sufficient yields of the 200 bp amplified product and analysed on a 2% agarose gel following staining.

**Figure 3:**
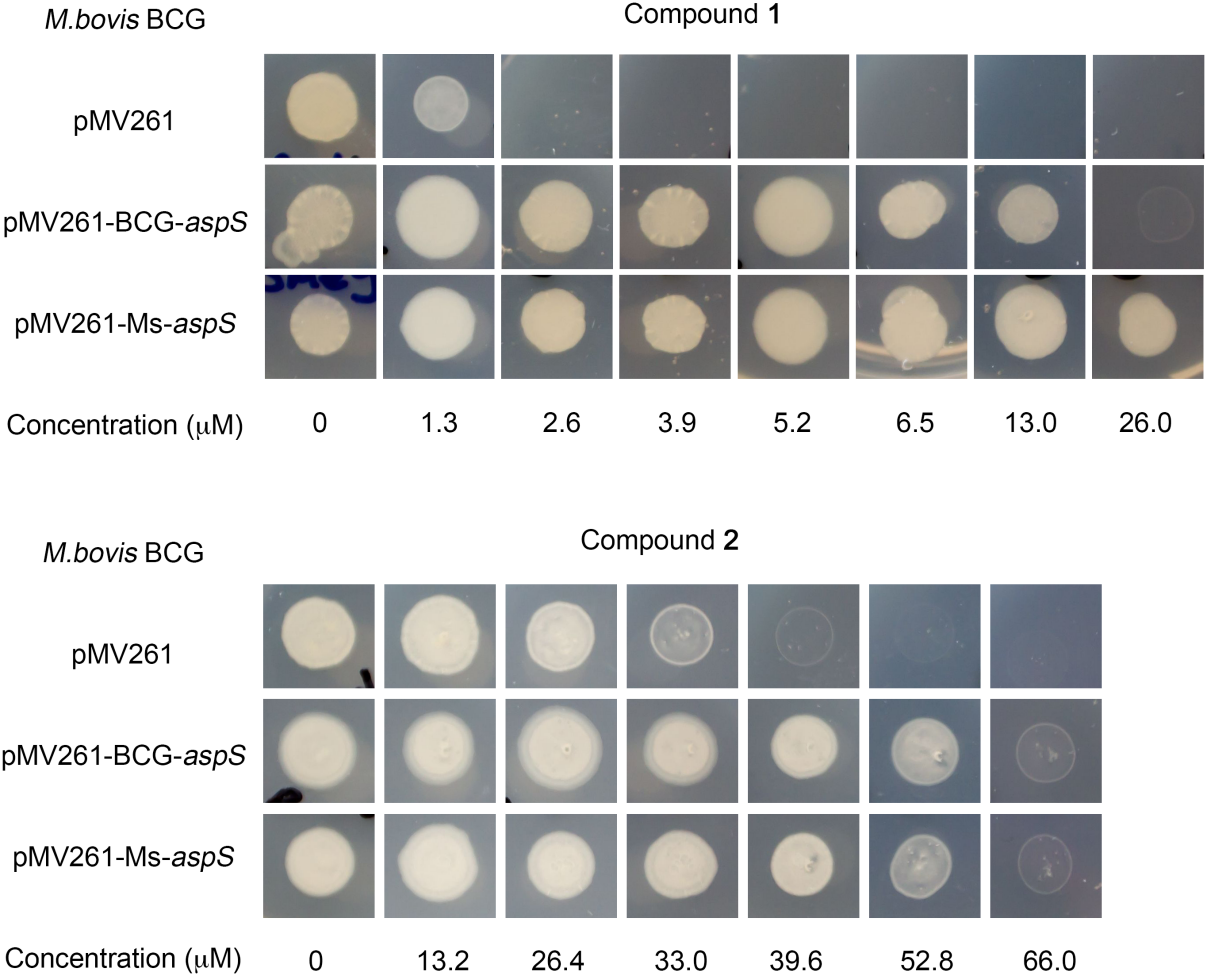
Whole cell target based resistance using overexpression of aspS in *M. bovis* BCG against compound 1 and 2. The overexpression plasmids pMV261, pMV261-BCG aspS and pMV261-Ms aspS were electroporated into M. bovis BCG with kanamycin at 25 µg/ml, and the MIC and resistance against compound **1** and **2** was evaluated.

Compound **1**, which has a 4-thiazolidinone core, was identified as an inhibitor of *M. tuberculosis* in a previous phenotypic screen [26]. Resistant mutants were isolated that showed a 46-fold increase in MIC against *M. tuberculosis* in comparison to the wild type strain. WGS of three mutants identified single nucleotide polymorphisms resulting in the substitutions at F526L and T570I in Mt-AspS. Transfer of the F526L mutation to the parental strain *via* recombineering resulted in resistance to **1**, establishing whole cell target engagement for **1**. Given the anti-mycobacterial activity of compound **1** [26], we selected this and the structurally related compound **2** (**Figure 1**) for use in a detailed biochemical and structural characterization of mycobacterial AspS. Initially, we raised spontaneous mutants in *M. bovis* BCG at 10× MIC of compound **1** (MIC = 2.6 µM), which occurred with a frequency of 4×10^8^ cells. WGS analysis of two spontaneous resistant mutants revealed, in total, 10 high-quality single nucleotide polymorphisms when compared to the *M. bovis* BCG reference sequence. Eight of these were detected in the sequenced wild-type strain, and therefore are likely to have arisen during laboratory storage and passage and are unlikely to confer the resistance phenotype. The first of two remaining single nucleotide polymorphisms was only detected in mutant 1 at 100% allele frequency and was predicted to result in a non-synonymous mutation in *aspS* (encoding an aspartyl-tRNA synthetase), resulting in the amino acid substitution D179N (^535^GAC>^535^AAC) (**Table 2**). In the second mutant (**Table 2**), the only discriminatory variant present was a single base change located 97 nucleotide bases upstream of *aspS* (position 2862347), likely a promoter-up mutation at the −10 consensus, changing the sequence from 5′-TGCAAT-3 to 5′-TACAAT-’3. The 5′-TAXXXT-3′ sequence, where X is any nucleotide, is a highly conserved promoter region. There is 5′-TG-3′ one nucleotide base upstream of the −10 motif, which could compensate for the potentially poor wild-type promoter sequence [51]. This promoter-up mutation would explain the resistance to compound **1**, and would be analogous to promoter mutations observed for *inhA* and isoniazid resistance [52]. The differences in the gene expression between the promoter up mutant, *aspS* mutant and the wild type *M. bovis* BCG was determined by RT-PCR using BCG *aspS* specific primers. The levels of the transcript was higher in the promoter up mutant as opposed to wild type BCG and the *aspS* mutant (**Figure 2**). Overexpression of *M. bovis* BCG *aspS* and *M. smegmatis aspS* in *M. bovis* BCG resulted in resistance against **1** and **2** (**Figure 3**). *M. bovis* BCG *aspS* and *M. smegmatis aspS* overexpression resulted in a ten-fold and greater than ten-fold increase in MIC (from 2.6 µM for the pMV261 strain) against **1**, respectively (**Figure 3**). In contrast, when *M. bovis* BCG *aspS* and *M. smegmatis aspS* were overexpressed, a 1.6-fold increase in MIC (from 39.6 µM for the pMV261 strain) was observed for **2**.

**Table 2:** Single nucleotide polymorphisms detected in *M. bovis* BCG spontaneous resistant mutants.

*M. bovis* BCG chromosome^a^	Wild-type allele	Predicted aminoacid change	Region	Spontaneous resistant mutants	
				1.1.1.1. Mutant 1	1.1.1.2. Mutant 2
2861716	**G**ac	D179N	*aspS*	**A**acT	**G**ac-
2862347	1.1.1.3. 1.1.1	1.1.1.4. n/a-	Upstream of *aspS*	1.1.1.5. G-	1.1.1.6. A

a Genomic positions are relative to *M. bovis* BCG str. Pasteur 1173P2 chromosome (Genbank accession: NC_008769.1). Mutations are reported relative to *aspS* which is encoded on the reverse strand.

### Expression and purification of Mt-AspS

To generate Mt-AspS recombinant protein for HTS, the pET28b vector was used to clone and express the C-terminally His_6_-tagged Mt-AspS. The resulting protein was purified from the soluble fraction by a single-step Ni^2+^ chelate affinity chromatography procedure and gave a single band on SDS-PAGE. The yield of the purified protein was 6 mg *per* litre of culture and the enzyme preparation was stable when stored at −80°C for up to six weeks.

### Mt-AspS and hexokinase/glucose-6-phosphate dehydrogenase *in vitro* activity assays

**Figure 4:**
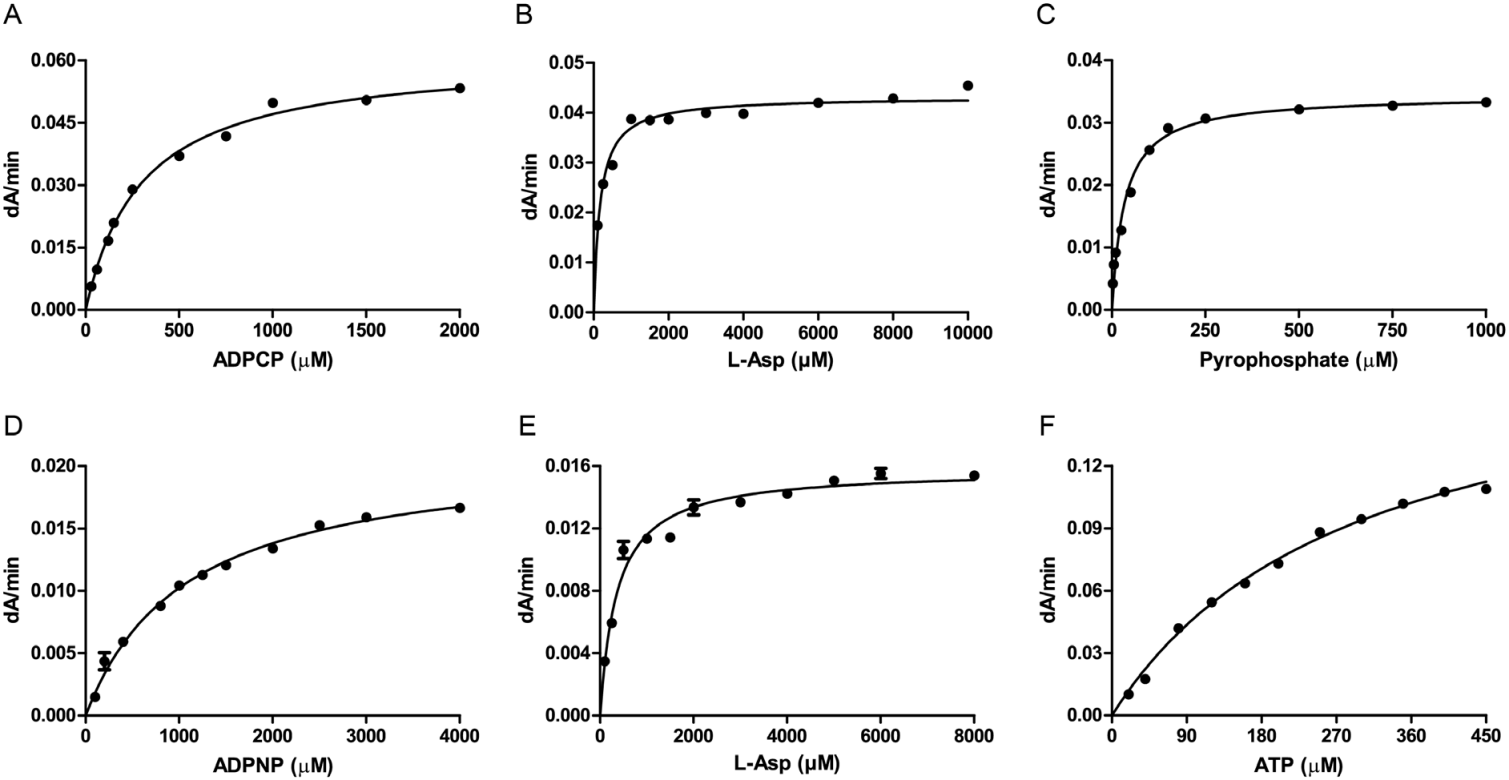
Substrate dependence of Mt-AspS activity. Michaelis-Menten curves were fitted for **A**) varying ADPCP as substrate. **B**) **L**-Asp as substrate at fixed saturating concentrations of ADPCP and PP*_i_.* **C**) PP*_i_* as substrate at fixed concentrations of ADPCP and **L**-Asp. **D**) ADPNP as substrate. **E**) **L**-Asp as substrate at fixed concentrations of ADPNP and PP*_i_*. **F**) ATP dependence of hexokinase/glucose-6-phosphate dehydrogenase activity. The initial velocity data (dA/min) were plotted against the substrate concentration. Each assay was done in triplicate and expressed as mean ± standard error of mean.

The tRNA-independent assays, monitoring either the fluorescence or absorbance signals, revealed Michaelis-Menten kinetics, displaying the prototypical hyperbolic saturation curve (**Figure 4A–E**) for the substrates ADPCP and ADPNP, as well as the co-substrates **L**-Asp and PP*_i_*, with K*_M_* values as reported in **Table 3**. Mt-AspS could utilise both ADPCP and ADPNP as substrates in the presence of the co-substrate **L**-Asp. The apparent K*_M_* for ADPNP (1077±74.83 µM) in the absorbance mode was 3.5 times higher than that for ADPCP (300.8±12.29 µM), indicating weaker binding of ADPNP. As expected, fluorescence mode assays were more sensitive than absorbance mode assays, but again ADPCP was the preferred substrate with a K*_M_* of 98.25±11.23 µM, 6-fold lower than the K*_M_* for ADPNP (604.7±40.27 µM). Thus, ADPCP was a better substrate in either assay mode and exhibited stronger binding as indicated by the lower K*_M_* value. The K*_M_* values obtained for **L**-Asp using ADPCP or ADPNP as co-substrates in the absorbance and fluorescence mode differed only by about one standard deviation (**Table 3**).

**Table 3:** Summary of K*_m_* determined for the Mt-AspS substrates and the hexokinase/glucose-6-phosphate dehydrogenase substrate.

AspS assay K*_M_* - absorbance mode			
Varied substrate	Unvaried substrate	K*_M_* (µM)	Correlation coefficient R^2^
ADPCP (30–2000 µM)	**L**-Asp (10 mM) and PP*_i_* (250 µM)	300.8±12.29	0.9949
**L**-Asp (100–1000 µM)	ADPCP (2 mM) and PP*_i_* (250 µM)	168.3±12.14	0.9669
PP*_i_* (2.5–1000 µM)	ADPCP (2 mM) and **L**-Asp (2 mM)	33.85±2.743	0.9842
ADPNP (50–4000 µM)	**L**-Asp (10 mM) and PP*_i_* (250 µM)	1077±74.83	0.9887
**L**-Asp (100–10000 µM)	ADPNP (3 mM) and PP_i_ (250 µM)	363.7±33.63	0.9685
**Hexokinase/Glucose-6-P dehydrogenase assay K** ***_M_*** ** - absorbance mode**			
**Varied substrate**	**Unvaried substrate**	**K** ***_M_*** ** (µM)**	**Correlation coefficient R^2^**
ATP (20–450 µM)	Glucose (10 mM) and NADP^+^ (500 µM)	293.1±17.10	0.9948
**AspS assay K** ***_M_*** ** - fluorescence mode**			
**Varied substrate**	**Unvaried substrate**	**K** ***_M_*** ** (µM)**	**Correlation coefficient R^2^**
ADPCP (7.5–1000 µM)	**L**-Asp (10 mM) and PP*_i_* (250 µM)	98.25±11.23	0.9544
**L**-Asp (25–5000 µM)	ADPCP (1 mM) and PP*_i_* (250 µM)	143.4±18.83	0.9450
PP*_i_* (2.5–1000 µM)	ADPCP (1 mM) and **L**-Asp (1.5 mM)	76.42±10.10	0.9648
ADPNP (25–2500 µM)	**L**-Asp (10 mM) and PP*_i_* (250 µM)	604.7±40.27	0.9912
**L**-Asp (25–5000 µM)	ADPNP (2.4 mM) and PP*_i_* (250 µM)	332.8±41.63	0.9478

We devised a hexokinase/glucose-6-phosphate dehydrogenase (HK/G6P-DH) coupling enzyme system with the substrates to measure the effect of **1** and **2** in the coupling enzyme system. A specific Mt-AspS inhibitor should not inhibit the coupling enzymes. In the fluorescence mode, it was not possible to determine K*_M_* of ATP, since we observed quenching at high concentrations of ATP. However, in the absorbance mode the HK/G6P-DH assay was suited for the determination of the K*_M_* value of ATP, which was 293.1±17.10 µM as calculated form non-linear least-squares fitting from the Michaelis-Menten plots (**Table 3** and **Figure 4F**).

### Determination of the IC_50_ of inhibitors

**Figure 5:**
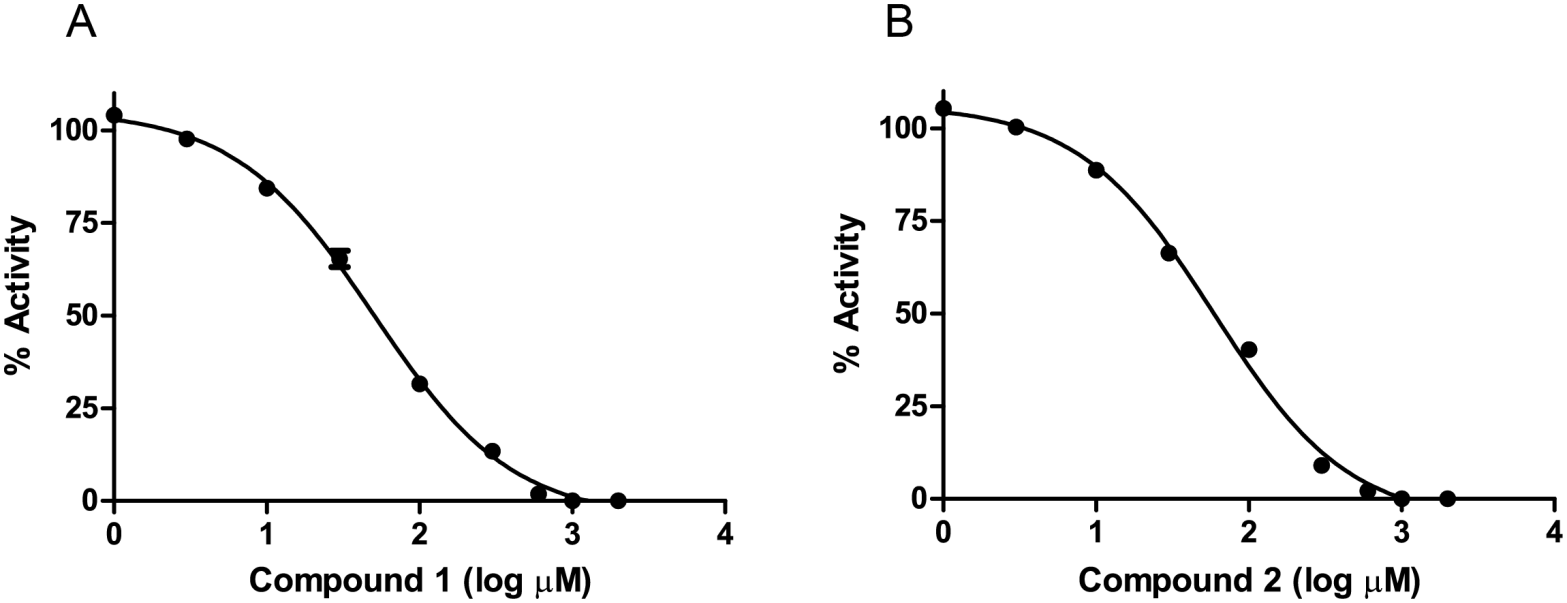
Dose response *in vitro* activity curves of the effect of compound 1 (A) and compound 2 (B) on Mt-AspS. The reaction mixture was added to increasing amounts of **1** and **2**, and the reaction initiated with the substrate PP*_i_* and the fluorescence intensity read using a microtitre plate reader. The assays were performed in triplicate and the percentage activity plotted *versus* the log µM concentration of **1** and **2**.

Inhibitor studies were performed using the substrates ADPCP and **L**-Asp at concentrations matching their K*_M_* values. Validation of the Mt-AspS assay using **1** and **2**, as well as in conjunction with the HK/G6P-DH coupling enzyme activity was performed as described in the materials and methods section. The Mt-AspS activity was plotted against inhibitor concentration and IC_50_ values were determined (**Figure 5**). At high concentrations, both **1** and **2** showed a tendency to aggregate when added to the aqueous reaction mixture. We therefore used the zwitterionic detergent CHAPS at 1.5% to counter aggregation of compounds as reported previously [53]. Both compound **1** and **2** retained inhibitory activity at approximately three times the critical micelle concentration (CMC) of CHAPS (CMC: 0.4920 to 0.6150%). Compound **1** inhibited Mt-AspS activity with an IC_50_ of 49.9 µM and a Hill slope of −0.9557 (R^2^ 0.9970) and compound **2** had an IC_50_ of 59.1 µM and a Hill slope of −0.9832 (R^2^ 0.9960). Both inhibitors turned out to be general promiscuous inhibitors and compound **1** showed 84.3% residual activity in the HK/G6P-DH coupling system assay at 300 µM concentration and compound **2** showed 87.5% activity at 100 µM concentration and exhibited steep dose response curves.

Compound aggregates are known to disrupt bacterial membranes and are responsible for inhibiting many enzymes at higher concentrations but could have biological activities at lower concentrations [54]–[56]. MIC values reported recently for compound **1** against *M. tuberculosis* mc^2^ 7000 were 0.7 µM in liquid media and approximately 18-fold higher (12.5 µM) in solid media [26]. Promiscuous compounds tend to be associated with a logP of 2.5 to 3 [57]. Indeed, with a logP value of 2.99, compound **1** falls within the logP-range of promiscuous compounds [26].

### Crystal structure of *apo* Ms-AspS and mapping of the inhibitor binding site

**Figure 6:**
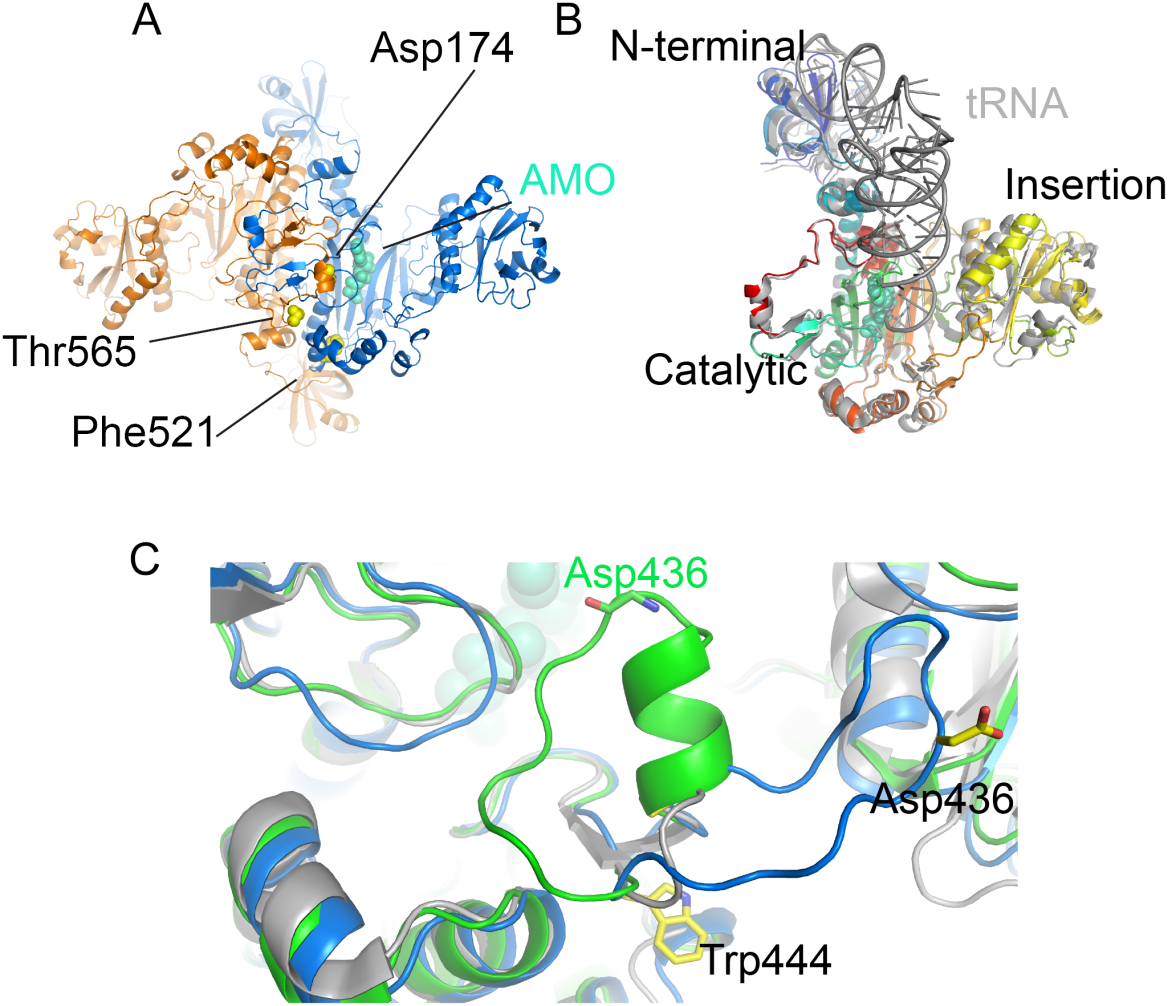
Structure of *M. smegmatis* AspS. **A**) Dimer of Ms-AspS in ribbon representation (colored by monomer) with resistance-conferring mutation sites indicated in yellow spheres. The binding site of aspartyl adenylate (AMO, spheres in cyan) is derived from the secondary structure-matched superposition with the structure of *E. coli* AspRS (PDB entry 1C0A, [61]). **B**) Superposition of the Ms*-*AspS monomer with the tRNA-bound structure *E. coli* AspRS (grey ribbon, PDB entry 1C0A). **C**) Detail of the superposition of Ms-AspS (blue ribbon), PDB entry 4O2D (green ribbon) of the same protein and *E. coli* AspRS (grey ribbon), focusing on the flexible loop spanning residues 427 to 444. To illustrate the variable conformation of the loop, sticks indicate the spatial positions of Trp444 and Asp436.

**Figure 7:**
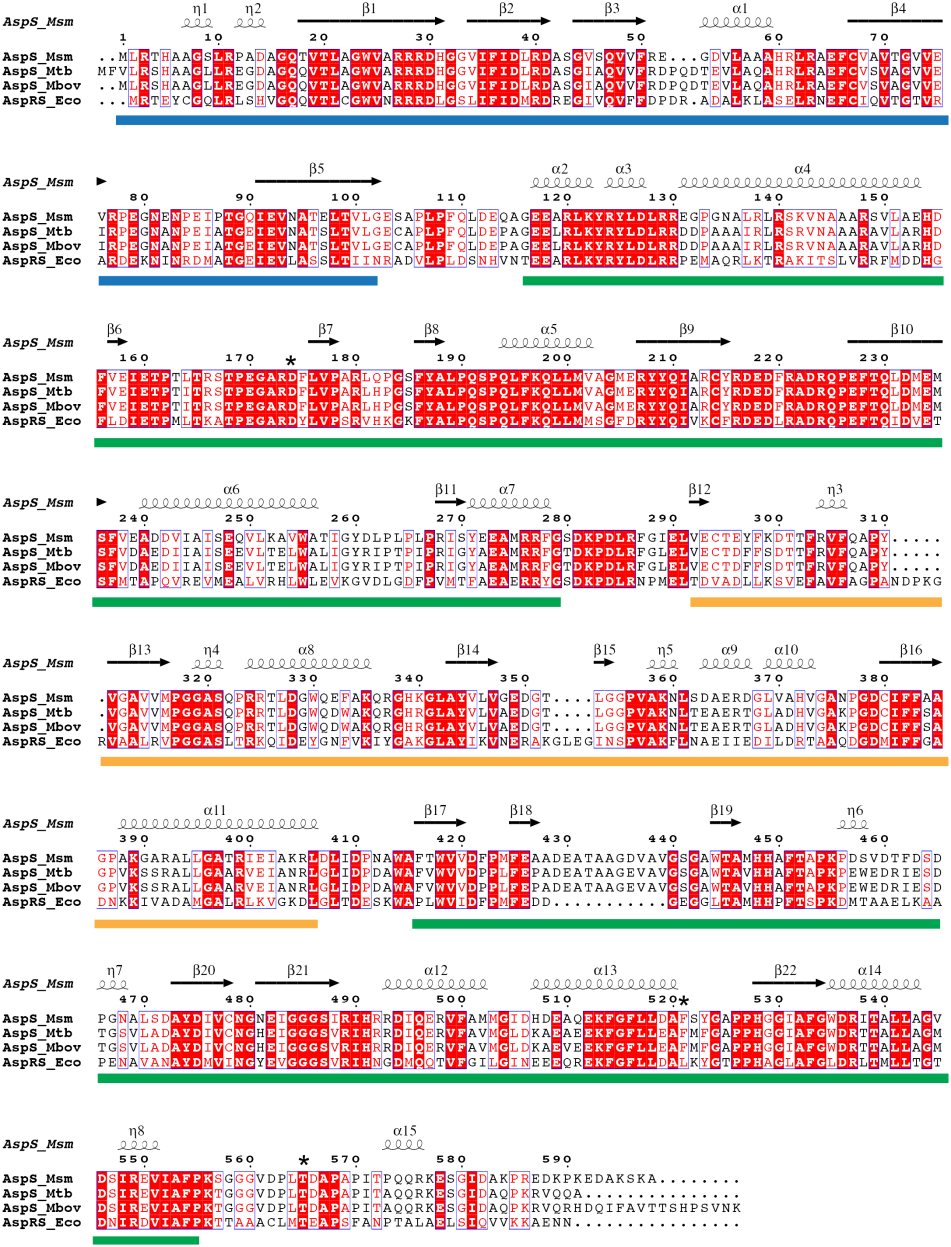
Alignment of the amino acid sequences of AspS of *M. smegmatis* (Msm), *M. tuberculosis* (Mtb) and *M. bovis* (Mbv) with that of *E. coli* AspRS (Eco). Bars in blue, green and orange indicate the N-terminal, catalytic and insertion domains of AspS, respectively. Asterisks indicate the sites of resistance-conferring mutations.

To aid future hit-to-lead medicinal chemistry efforts, we determined the crystal structure of Ms-AspS by molecular replacement, refining the model to a resolution of 2.4 Å (**Table 4**). As the closely related aspartyl tRNA synthase AspRS from *E. coli* [51], Ms-AspS forms a dimer (**Figure 6A**). In the context of the crystal lattice, the two monomers are related by crystal symmetry burying an extensive dimer interface from solvent. The tertiary structure is conserved across species boundaries, with a 3-domain architecture consisting of the N-terminal, β-barrel like domain, a central catalytic domain and an insertion domain that is spliced into the sequence of the catalytic domain (**Figure 7**). The superposition with the tRNA-bound structure of *E. coli* AspRS (**Figure 6B**) illustrates the close structural relationship between the two enzymes (48% sequence identity). Despite the fact that the Ms-AspS enzyme was crystallised without tRNA, the orientation of the N-terminal domain, which mediates anticodon recognition, and the insertion domain, which contributes to positioning the tRNA in the active site pocket, relative to the catalytic domain barely changes (**Figure 6B**). In the vicinity of the active site, Ms-AspS includes an extended flexible loop encompassing residues 429 to 441 (**Figure 6C**). The corresponding loop in *E. coli* AspRS is 11 amino acid residues shorter (**Figure 7**). An *apo*-structure of Ms-AspS has recently been deposited in the PDB (entry 4O2D), in which this loop assumes a very different conformation, forming a 2-turn α-helix and folding back onto the aspartyl adenylate binding site. The differential conformation likely occurs due to differences in crystal packing constraints. The two coordinate sets have different space group symmetry (*P*2_1_2_1_2_1_ and *C*222_1_, respectively), and in the present structure, the conformation of the 429–441 loop is constrained by forming contacts with a symmetry-related copy of MsAspS. In contrast, the packing environment in 4O2D imposes no direct constraints on the conformation of this loop.

**Table 4:** Statistics of X-ray diffraction data and crystallographic structure refinement.

X-ray diffraction data	
Crystal	Ms-AspS
X-ray source	Rigaku MM007HF
Wavelength (Å)	1.5418
Space group	*C*222_1_
Cell parameters *a,b,c* (Å)	71.6, 141.5, 156.8
Molecules per asymmetric unit	1
Resolution, last shell (Å)	42.04–2.40 (2.53–2.40)
*R_merge_* (%)^1^	12.5 (65.4)
Total, unique observations	518,021, 31,382
*I/σ(I)* 1	25.1 (5.1)
Completeness (%)^1^	99.7 (98.3)
Multiplicity^1^	16.5 (15.6)
**Refinement**	
Resolution range (Å)	78.41–2.40
Unique reflections used	29,771
*R_cryst_,* R_free_ (%)	21.5, 24.6
Number of non-hydrogen atoms	4,456
Protein	4,320
Solvent	136
RMSD bonds (Å)	0.007
RMDS angles (°)	1.2
B-factors	
Wilson B-factor (Å^2^)	38.8
Overall average (Å^2^)	34.1
Protein average (Å^2^)	34.4
Solvent average (Å^2^)	23.5
RMSD B-factors	0.8
Ramachandran plot^2^	
Favoured region (%)	96.5
Allowed regions (%)	3.5
Disallowed (number)	0

1 Numbers in parentheses refer to the last shell. ^2^Ramachandran statistics were determined using Molprobity [62].

**Figure 8:**
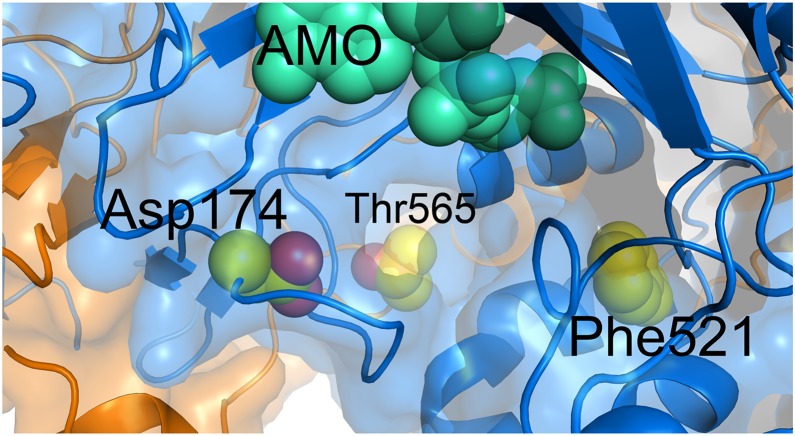
Molecular surface of Ms-AspS showing the inhibitor-binding pocket adjacent to the aspartyl adenylate binding site, showing the position of the aspartyl adenylate donor substrate relative to the proposed pocket. The sites of resistance conferring mutations are indicated by spheres in yellow, and the aspartyl adenylate donor substrate (AMO), positioned according to the superposition with *E. coli* AspRS (PDB entry 1C0A, [61]) is indicated by spheres in turquoise.

We mapped the location of mutations conferring resistance, reported previously [26] and generated here, to **1** on the present structure. When considered in the context of the Ms-AspS dimer, the three resistance mutations are in close proximity of each other, surrounding a narrow pocket that opens to the aspartyl adenylate binding site in the catalytic domain (**Figure 8**). Thr565 (*M. tuberculosis* Thr570) is contributed by the opposing monomer and sits at the bottom of the pocket. Asp174 (*M. tuberculosis* Asp179) lines the side of the pocket, while Phe521 (*M. tuberculosis* Phe526) forms close contacts with residues that do line the pocket.

**Figure 9:**
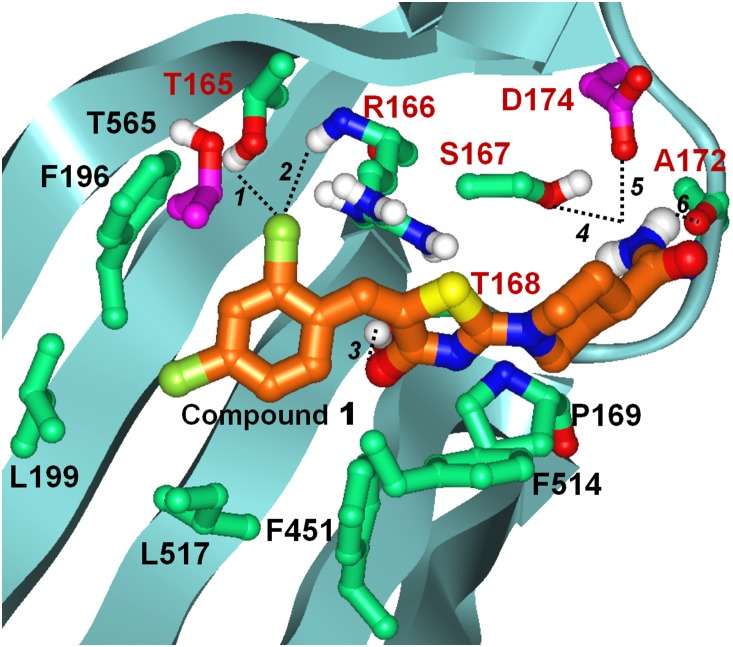
Induced Fit docked pose of compound 1 at the *M. smegmatis* AspS crystal structure. Only amino acids forming direct interactions with compound **1** are shown. Carbon atoms of AspS are colored light green except for carbons of resistance conferring mutant site residues, which are colored purple. Carbons of compound **1** are shown in orange; all other atoms are colored by atom type (Oxygen red, Nitrogen blue, Sulphur yellow, Chlorine dark green, Hydrogen white). Labels of residues participating in polar interactions are colored brown, labels of all other residues are in black. Polar interactions are indicated with dashed lines and are numbered. Distances between heavy atoms of the atoms groups participating in polar interactions are as follows: *1*. 3.1 Å; *2*. 3.8 Å; *3*. 3.0 Å; *4*. 3.2 Å; *5*. 3.1 Å; *6*. 3.7 Å.

Using Induced Fit docking protocols we docked compound **1** into this pocket (**Figure 9**). The obtained docked pose predicts a number of favorable non-polar and polar contacts with residues lining the pocket as follows. The piperidine ring in compound **1** participates in non-polar interactions with P169 while the thiazolidinone moiety forms aromatic interactions with F514. The dichlorophenyl ring has non-polar/steric contacts with a number of residues: F451, L517, L199, F196, as well as T565 from the adjacent subunit. The side chain of T565 is in close proximity of the ligand’s phenyl ring (the closest ring carbon is at 3.8 Å from the threonine side chain methyl). Guided by the resistance conferring mutation sites that include this threonine, we generated the T565I AspS mutant structure *in silico* and attempted to re-dock compound **1** using the same protocols, however, no successfully docked poses could be obtained, consistently with the results of the resistant mutations study. Another resistance conferring mutant site corresponds to D174 which is the residue predicted to form hydrogen bonding interactions with the amide group of compound **1** (**Figure 9**). Re-docking compound **1** into the D174N mutant AspS structure resulted in a different positioning of the piperidine-amide that has no direct interaction with residue 174 (not shown). In this pose the distance between the amide groups of the N174 side chain and compound **1** is 8.3 Å. Thus, docked poses of compound **1** at the D174N mutant and the wild type *M. smegmatis* AspS structure are consistent with this residue being an important contributor to the binding of compound **1** at AspS. The docking hypothesis of compound **1** at the AspS crystal structure predicts several favorable polar contacts also, as follows. In addition to hydrogen bonding with D174, the amide group of compound **1** forms hydrogen bonding interactions with S167 and the backbone carbonyl of A172. The carbonyl of the thiazolidinone participates in hydrogen binding with T168 while the 2-chlorine substituent on the phenyl forms polar interactions with T165 and the backbone amine of R166. While F521 is not directly accessible to bound ligands, it is possible that the resistance-conferring mutation of F521 to Leu induces conformational changes that propagate to the proposed pocket. While we attempted to generate crystals of Ms-AspS bound to **1** and **2**, we could not overcome poor solubility in the crystallization buffers. In the absence of co-crystallized ligand, the proposed docked pose of compound **1** provides a plausible model of its Asp-bound structure with a number of favorable polar and non-polar contacts as described here.

## Conclusion

The phenotypic antitubercular activity of the 4-thiazolidinones has been known for several years based on PubChem deposition of HTS results for several libraries totaling over 300,000 compounds against virulent *M. tuberculosis* [7]–[9]. In a continuation of our work to identify targets of interesting and novel active samples from the TAACF screens, we were pursuing a TAACF active **1**, a member of the 4-thiazolidinones. We reasoned that compounds active against the whole bacillus *in vitro* would offer advantages for hit-to-lead development, as they are already active against the bacterium *in vitro*. Starting with scaffolds that show antibacterial activity offers significant advantages over pure target-based optimization programs in that optimization starts from the privileged position of proven whole-cell antibacterial activity [11]. Nevertheless, before initiating a rational drug discovery and optimization program it is crucial to identify a putative target as well as a crystal structure of that target. A recent publication has identified a potential target as *M. tuberculosis* aspartyl-tRNA synthetase AspS for analog **1** [26]. Our work with both **1** and **2** further validate AspS as the putative target of the 4-thiazolidinones. Furthermore, we have solved the *apo*-structure of the target and developed a binding model for this inhibitor class that should help produce more rationally designed inhibitors to improve binding and inhibition. For example, it is interesting to note that **1** is a superior inhibitor to compound **2** although the differences are relatively modest. As well as being a better inhibitor, possibly due to specific hydrogen bonding of the carboxamide in the active site, **1** should be more hydrolytically stable than **2** (a carboxamide versus a carboxylic acid ethyl ester). In fact, it is likely that hydrolysis of the ester to the free –COOH might significantly alter protein binding and inhibition due to the neighboring aspartic acid residue (Asp174/Asp179) lining the proposed ligand binding site, potentially leading to charge-charge clashes. These possibilities suggest new analogs that might optimize favorable charge-charge interactions in the protein, and we are currently working on new analogs to improve solubility and binding with the goal of obtaining better inhibitors that might allow co-crystallization. It should be pointed out, however that both **1** and **2** fall into thiazolidinone or rhodanine class that are broadly considered Pan Assay INterference compounds or PAINS as they show up as promiscuously active in numerous HTS assays and are notoriously difficult to optimize against screening targets [58]. In fact, there is a general move to eliminate the class from screening libraries and proscribe reports of these compounds as assay hits in the literature [58], [59] in spite of the fact that several known drugs are based on the scaffold that include ralitoline, etozoline, pioglitazone and thiazolidomycin [60]. Our data reported herein support the likely specific activity of compounds 1 and 2 against AspS as resistant mutants arose specifically to this protein at a specific locale in the target identified. Typically, broadly acting, promiscuous compounds that have numerous targets do not support target identification *via* generation of resistant mutants since target cells are unable to generate effective multiple target mutations that bypass blockades against several targets. When resistance does arise to these types of compounds, it typically comes *via* entry and exit proteins either by restricting drug access or active efflux of the agent. From these (PAINS) compounds we have identified a druggable protein target via specific mutations, produced a crystal structure and binding model, and developed a HTS assay for further screening of diverse chemical libraries. Hence, at least in the case of **1** and **2** useful information and research has accrued speaking to their value as chemical probes. We would also note, however, that future research and screening in an *in vitro* Mt-AspS and HK/G6p-DH assay developed through this work and adapted to a 96 well plate format suitable for HTS screening of compound libraries will be utilized to identify alternative, improved scaffolds that pass PAINS filters and are suitable for hit-to-lead medicinal chemistry programmes to generate more soluble and target specific inhibitors.
